# Mental Dietetics:—The Effects of Stimulants, Solid and Fluid, on the Mind
*The Use of Alcoholic Liquors in Health and Disease: Prize Essay. By William B. Carpenter. London, 1850.Temperance and Total Abstinence, or the Use and Abuse of Alcoholic Liquors in Health and Disease. By Spenser Thomson, M.D. London, 1850.


**Published:** 1851-01-01

**Authors:** 


### Art. V.—MENTAL DIETETICS:—THE EFFECTS OF STIMULANTS, SOLID AND FLUID, ON THE MIND.*

The human mind is never in a state of absolute repose. Impulses from within and influences from without continually disturb its equanimity. Inactivity is incompatible with thought. The active principles of our nature, as Dugald Stewart and other philosophers describe them; our appetites and desires, our affections and passions, our hopes, and "fears which kindle hope," are constant sources of emotion, resembling those natural springs and under-currents which unceasingly agitate the surface of a lake. These impulses originate in the mind itself; they are emanations, strictly speaking, from within, giving rise to a succession of thoughts and feelings variously modified, according to the peculiar idiosyncracy of each individual. There are other influences, however, operating from without, which take their rise from the body, for our

* The Use of Alcoholic Liquors in Health and Disease: Prize Essay. By William B. Carpenter. London, 1850.

Temperance and Total Abstinence, or the Use and Abuse of Alcoholic Liquors in Health and Disease. By Spenser Thomson, M.D. London, 1850.

spiritual is so intimately blended with our material nature, that a reciprocal interchange of impressions is constantly taking place. Every change of organic function is followed by a corresponding change in our mental frame; and in like manner, the lights and shadows which flit across the mind excite or depress the energies of our nervous system. This mysterious influence of the mind upon the body has been poetically compared to that which the sun was supposed to exercise on the Egyptian statue of Memnon, which was said to become musical when irradiated with its beams. The mode in which two entities, so essentially different from each other, are united, the link of connexion between them, is utterly unknown; but this much is certain, that their mutual harmony gives rise to that state of health which communicates a certain degree of tone and buoyancy to all our feelings, which Shakespeare admirably describes when he makes Romeo say—

> "My bosom's lord sits lightly on his throne,
> And all this day an unaccustomed spirit
> Lifts me above the ground with cheerful thoughts."

The consciousness of health is something more than mere freedom from pain; it is a positive pleasure.

"The pleasure which attends good health," observes Dr. Thomas Brown, "like every other long-continued bodily pleasure, we may suppose to be diminished by habitual enjoyment; and it is therefore chiefly on recovery from sickness, when the habit has been long broken by feelings of an opposite kind, that we recognise what it must originally have been. To those who know what it is to have risen from the captivity of a bed of sickness, I need not say that every function is more than mere vigour; it is a happiness but to breathe and to move; and not every limb merely, but almost every fibre of every limb, has its separate sense of enjoyment."*

This happy state of mental and bodily health; this unity, and combined harmony, constituting all that can be conceived pleasurable in the "voluptas vitæ," may be deranged by a variety of the causes referred to, operating from within or from without; the former comprehending those changes which originate in the mind itself, the latter those which arise from certain conditions of the body. The influence of climate; the state of the atmosphere, the quantity of its electricity or moisture; its barometrical pressure and various alternations, operate sensibly upon the feelings and mental powers of every person; how much does its purity exhilarate, how much its contrary condition depress the mind! What a blessed thing it is to breathe fresh air, exclaimed Count Struensee,

* Lectures on the Philosophy of the Human Mind. By Thomas Brown, M.D. 4 vols. Edinburgh, 1820. Vol. i. p. 396.

upon quitting his dungeon, although he was being led out to the place of his execution. The effect of different aliments, solid and fluid, upon the mind; more especially those which are of a stimulating description, is still more striking; and these we now purpose considering, under the head of Mental Dietetics.

The physiology of digestion—the mastication and insalivation of food and its propulsion into the stomach; its conversion into chyme, and the course of the vessels by which its nutritious particles are taken up in the form of chyle, and poured into the current of the venous blood just as it is reaching the right side of the heart, and the transmission of this venous blood, mixed with the chyle, into the lungs, there to be arterialized and rendered fit for the nourishment and support of every part of the body, need not here be described; suffice it to observe, that the great end of the digestive function is to supply the vital stream with the elements of nutrition. The process of digestion, therefore, in reality, terminates in the lungs. The object of dietetics, as a legitimate branch of psychological as well as physiological science, is to regulate the supply of such food, whether taken in a solid or fluid form, as will be most easily digested. The very great proportion of blood which is sent directly to the brain, and which Haller states to be one-fifth of the whole current, would lead us, *à priori*, to anticipate that this organ would be the first to suffer from any morbid alteration in the condition of the circulating fluid. But this is not all; we must remember that an immediate connexion is established between the brain and the digestive organs, by means of the eighth pair of nerves and the sympathetic system; and hence Mr. Mill, in his analysis of the phenomena of the human mind, correctly observes,

"Connexions are proved to exist between our ideas and certain states of these organs. Thus anxiety, in most people, disorders the digestion. It is no wonder, then, that the internal feelings which accompany indigestion should excite the ideas which prevail in a state of anxiety. Fear in most people accelerates, in a remarkable manner, the vermicular motion of the intestines. There is an association, therefore, between certain states of the intestines and terrible ideas; and this is sufficiently confirmed by the horrible dreams to which men are subject from indigestion; and the hypochondria, more or less afflicting, which almost always accompanies certain morbid states of the digestive organs. The grateful food which excites pleasurable sensations in the mouth, continues them in the stomach, and as pleasures excite ideas of their causes, and these of similar causes, and causes excite ideas of their effects, and so on, trains of pleasurable ideas take their origin from pleasurable sensations in the stomach. Uneasy sensations in the stomach produce analogous effects. Disagreeable sensations are associated with disagreeable circumstances; a train is introduced in which one painful idea following another, combinations to the last degree afflictive are sometimes introduced, and the sufferer is altogether overwhelmed by dismal associations."*

To the same causes, viz., reciprocity between sensations and ideas, may be ascribed those instinctive principles of action which originate in certain wants that are common to all animals.

"Our appetites (says Dugald Stewart) are three in number, hunger, thirst, and the appetite of sex. Of these, two were intended for the preservation of the individual, the third for the continuation of the species; and without them reason would have been inefficient for those important purposes. Suppose, for example, that the appetite for hunger had been no part of our constitution; reason and experience might have satisfied us of the necessity of food for our preservation—but how should we have been able, without an implanted principle, to ascertain according to the various states of our animal economy the proper seasons for eating, or the proper food that is salutary to the body? The lower animals not only receive this information from nature, but are, moreover, directed by instinct to the particular sort of food that is proper for them to use in health and in disease."†

So also Dr. Reid observes:

"Though a man knew that his life must be supported by eating, reason could not direct him when to eat, or what; how much or how often. In all these things appetite is a much better guide than reason. Were reason only to direct us in this matter, its calm voice would often be drowned in the hurry of business and charms of amusement. But the voice of appetite rises gradually; and at last becomes loud enough to call off our attention from any other employment."‡

We must not, however, restrict our observations to the means which an all-wise Providence has provided for suggesting, instinctively, the means of gratifying physical desires; the psychologist must go further. In a state of nature, doubtless, instinct would be an unerring guide; and yet, so strange are the effects of habit, that the appetite of animals has been changed by domestication, and some brought to subsist on food unadapted even to their organic structure. Herbivorous and graminivorous animals have been so trained as to live on animal food; and carnivorous animals, the lion, tiger, cat, &c., been taught to live, and thrive tolerably, on a vegetable diet. Horses, on the coast of Arabia, are constantly fed upon fish, herbage being deficient; and Bishop Heber informs us that in Norway, as well as in some parts of Hadramant and the Coromandel coast, they are constantly fed on the

* Analysis of the Phenomena of the Human Mind. By James Mill. 2 vols. 8vo. London, 1829. Vol. i.

† The Philosophy of the Active and Moral Powers of Man. By Dugald Stewart. 2 vols. Edinburgh, 1828. Vol. i.

‡ Essays on the Powers of the Human Mind. 3 vols. Edinburgh, 1812. Essay iii. c. 50.

refuse of fish. In the Philosophical Transactions, Dr. Tyson states that a horse in London, accustomed to stand at a tavern door where oysters were left, acquired a liking for this strange food—crunched the shells, and swallowed them with their contents. In Monro's narrative relating to the coast of Coromandel, we learn that the dealers fatten their horses by giving them balls composed of the boiled flesh of sheep's heads, mixed with grain. A lamb, during a long sea-voyage, was induced to live upon animal flesh; and afterwards, upon being turned into a pasture, refused to crop the grass destined for its support. Even a young wood-pigeon has been brought to relish flesh, and refuse every other kind of diet. The effects of these different kinds of food on the temper and disposition of such animals are curious. It has been remarked, upon good authority, that herbivorous animals, which are trained to eat flesh, become extremely ferocious; and that carnivorous animals fed upon a vegetable diet, become, on the other hand, extremely gentle. We are informed by Bishop Heber, that the cattle which are fed upon fish fatten rapidly; but the diet changes their nature, and renders them unmanageably ferocious. It has been observed, too, that the Tartars and other nations which live principally on animal food are warlike and cruel, while the Brahmin and Gentoo, subsisting chiefly on vegetable diet, evince a peaceable and mild disposition. Fuseli, the painter, was in the habit of eating raw meat, for the purpose of engendering in his imagination horrible fancies. Dryden, it is said, did the same. It is related that Mrs. Radcliffe, when writing the "Mysteries of Udolpho," took heavy suppers, and ate uncooked meat. Voltaire humorously suggests, that Cromwell must have been labouring under a fit of indigestion, when the idea of bringing Charles I. to the scaffold entered his mind. When Lord Byron, in one of those capricious episodes of his life which he frequently indulged in, entertained a horror of corpulency, he would accustom himself, for days, to live on biscuits and soda water; and after this fit of abstemiousness was over, a dinner of beef-steaks was wont to make him savage. He would then argue, that eating flesh excited men to war and bloodshed:

> "Hence Pasiphæ promoted breeding cattle,
> To make the Cretans bloodier in battle."

His biographer says, "one day as I sat opposite to him, employed, I suppose, rather earnestly over a beef-steak, after watching me for a few seconds, he said in a grave tone, 'Moore, don't you find eating beef-steaks make you ferocious?' "* The zeal with which Shelley, at one period of his life, denounced the use of animal food, and the cruelty of

* Life and Works of Lord Byron. By Thomas Moore. 17 vols. Murray, 1830. Vol. iv. p. 158.

slaughtering animals, may be remembered. In the Utopian perfection of the ideal world revealed by "Ianthe," in the poem of Queen Mab, he describes a meat diet as giving rise to "the germs of misery, death, disease, and crime;" and depicts man, when regenerated, living entirely on the fruits of the earth:

> "No longer now
> He slays the lamb that looks him in the face,
> And horribly devours his mangled flesh."

The history of mankind sufficiently evinces, that not only individuals, but even nations, have lived almost exclusively on vegetable food; it has, however, been abundantly proved, that a mixed diet is the most conducive to health: nevertheless, there are certain conditions of the system, when the physician is called upon to substitute a purely vegetable for a mixed animal diet, and, *vice versâ*, a stimulating animal for a vegetable regimen. In Watt's work on Diabetes, the case is detailed of a student who had been the subject of severe depletory measures, and whose intellect was raised in the inverse proportion to the reduction of his body.

"From my own observation," observes Dr. Thackrah, "I am convinced that by a low diet the intellect is often relieved and invigorated; but, on the other hand, I have seen it improved by an opposite system. A young gentleman who has tried various diets, assures me that his mind is most capable of exertion when his body is most plentifully nourished. He now takes flesh twice, and milk once a day. Dr. Stark, on taking beef after a low diet, had 'a keenness for study.' It is evident, from these and similar observations, that the intellect may be powerful on contrasted diets, and that advantage has been derived from an increased proportion of flesh, as well as from the opposite change. To vigour of mind, a proper circulation through the brain, a supply of blood healthy both in quality and volume, is always requisite. Now the digestive organs of individuals vary so much in their power of abstracting nourishment from like qualities and kinds of aliments, that a diet which is moderate to one will be excessive to another. One student, living chiefly on vegetables, may have the brain weak or torpid from the defect of good blood; another, taking a richer diet, may have the brain oppressed from sanguineous excess. Let there be a mutual transfer of diet to suit the opposite constitutions of their digestive organs, and the state of the brain in both individuals will be materially improved."*

The principles upon which the physiology of digestion is explained must be clearly kept in view before we can appreciate these facts psychologically, for in pursuing, step by step, the induction which enables us to connect the changes which take place in the body with

* Lectures on Diet and Digestion. By Charles Turner Thackrah. London, 1824. p. 64.

mental phenomena, we must trace the mode in which these various elementary substances are introduced into the system, and the influence which every stage of this process has upon the mind. When the stomach has received a certain supply of food, its bloodvessels and nerves are stimulated to increased activity, whence arises an afflux of blood into its internal lining membrane, accompanied by a copious secretion of gastric juice. This great afflux of blood, which takes place in the stomach and intestines, diminishes the quantity of circulating fluid on the surface and in other distant parts of the body, and is accompanied by an increase of nervous energy, concentrated from the centres of the nervous system. Hence, delicate persons frequently experience a certain degree of coldness, or chill, after taking a full meal; and most people feel disposed, after dinner, to draw near to the fire. This, however, is not all: the distended stomach curves round upon its axis, and presses upon the descending aorta, which impedes the downward current of the circulation. Hereby the blood accumulates in the vessels of the head; the face is observed to be unusually flushed; and sometimes the action of the lungs being interfered with, occasions for awhile difficulty of breathing and a sense of oppression in the region of the heart. This condition fully accounts for the impaired intellectual activity which is experienced after a plentiful repast, and, accordingly, the whole animal creation evinces a disposition to remain quiet and sleep after eating. The lion, after gorging himself to repletion, "smoothes his brindled mane," and lies down in his forest solitude in a state of sluggish repose;—the wolf, with his hide distended, betakes himself to his mountain-fastness; and slumbers unconsciously until his system becomes again reduced, when, urged by the pangs of hunger, he again goes forth, almost wasted to a skeleton, howling in search of prey; the boa-constrictor, gorged to excess, remains in a state of stupor many days, and, until digestion has performed its office, cannot rouse itself to activity. So is it with the gourmand: he, too, sinks into a profound and stertorous sleep, and not unfrequently falls a victim to apoplexy. When, however, the stomach has relieved itself of its burthen,—when it diminishes in bulk, and no longer presses on the aorta,—the respiration becomes free,—the circulation is again restored,—the nervous energy becomes equably diffused through the system, and the intellectual faculties recover their accustomed vigour. A curious observation is made by Dr. Paris: he says that, at the moment the chyle is poured into the blood, "the body becomes enlivened; then it is that animals are roused from that repose into which they had subsided during the earlier stages of digestion, and betake themselves to action—then it is that civilized man feels an aptitude for exertion."* We believe,

* A Treatise on Diet. By J. A. Paris, M.D. 2nd Edition. London, 1827. p. 111.

however, the relief is more immediate than this, and is experienced as soon as the stomach is partially unburthened, otherwise, considering the period which various aliments take to be digested, man would, after eating, be much longer than he is, indisposed to activity.* The nutritive particles of the food being thus, in the form of chyle, mixed with the blood, and supplying it with the elements which enable it to repair the waste of the animal system, it is obvious that the health both of the body and of the mind must depend on the quality and quantity of the vital stream. According to Lecanu, the proportion of the red globules of the blood may be regarded as a measure of vital energy, for the action of the serum and of the globules on the nervous system is very different. The former scarcely excites it, the latter do so powerfully. Now those causes which tend to increase the mass of blood, tend also to increase the proportion of red globules; whilst those which tend to diminish the mass of blood, tend to diminish the proportion of the globules.† The result is obvious. A large quantity of stimulating animal food, without a proper amount of exercise, augments the number of the red globules, and diminishes the aqueous part of the blood. Hence the nervous system becomes oppressed, the brain frequently congested, and the intellectual faculties no longer enjoy their wonted activity. In the meantime, the system endeavours to relieve itself by throwing a counter-stimulus upon certain other organs, the functions of which are morbidly increased. The blood in such cases becomes preternaturally thickened, and its coagulum unusually firm. ‡ On the other hand, if the system be not supplied with the requisite amount of nutrition, the blood becomes, by the loss of its red corpuscules, impoverished in quality, and in cases of extreme abstinence diminished in quantity. In these cases the powers of the mind soon become enfeebled. "Among the poorer classes of children (observes Dr. Andrew Combe), the children as well as the

* From a Table drawn out by Dr. Beaumont, we learn that the mean time of digestion in the stomach (*or chymification*) of various articles of food, is as follows:—

| | H. M. | | H. M. |
|---|---|---|---|
| Roast beef | 3 30 | Roast turkey | 2 30 |
| Beef-steak, broiled | 3 0 | Roast pig | 2 25 |
| Salt beef, boiled | 2 45 | Venison steak | 1 35 |
| Boiled mutton | 3 0 | Fricasseed chicken | 2 45 |
| Boiled salt pork | 4 30 | Cheese, old and strong | 3 30 |
| Boiled fowl | 4 0 | Hashed meat | 2 30 |
| Roast fowl | 4 0 | Boiled tripe | 1 0 |
| Roast duck | 4 0 | Boiled eggs, soft | 3 0 |
| Boiled turkey | 2 25 | Boiled cabbage | 4 30 |

Experiments and Observations on the Gastric Juice. By W. Beaumont. Edinburgh, 1838. p. 37.

† Manual of Chemistry." By William Thomas Brande. 2 vols. London, 1848. Vol. ii. p. 1772.

‡ An Inquiry into the Nature and Properties of the Blood in Health and in Disease. By Charles Turner Thackrah. Edited by Thomas Wright, M.D. London, 1824. p. 192.

parents suffer much, both physically and morally, from insufficient food. Their diet being chiefly of a vegetable nature, and consisting of a scanty allowance of porridge, potatoes and soups, with very little butcher's-meat, is far from adequate to carry on vigorous growth in the young or repair waste in adults; hence arise in the former an imperfect development of the body, a corresponding deficiency of mental power, and a diminished capability of resisting the causes of disease. In work-houses and other charitable institutions, ample evidence of these deficiencies obtrudes itself upon our notice, in the weak and stunted forms and very moderate capacities of the children. Under an impoverished diet, indeed, the moral and intellectual capacity is deteriorated as certainly as the bodily; and full exposition of the fact, and of the principles involved in it, would be a great public benefit. When Sir John Franklin and his companions were reduced to a state of starvation at Fort Enterprise, in November, 1821, he was struck with the signs of weakness of intellect which they exhibited. I observed, says he, that as our strength decayed our minds exhibited symptoms of weakness, evinced by a kind of unreasonable pettishness with each other. Each of us thought the other weaker in intellect than himself, and more in need of advice and assistance. So trifling a circumstance as a change of place, recommended as being warmer and more comfortable, and refused by the other from dread of motion, frequently called forth fretful expressions, which were no sooner uttered than atoned for—to be repeated perhaps in the course of a few minutes. The same thing often occurred when we endeavoured to assist each other in carrying wood to the fire; none of us were willing to receive assistance, although the task was disproportioned to his strength. On one occasion, Hepburn was so convinced of this waywardness, that he exclaimed—'Dear me, if we are spared to return to England I wonder if we shall recover our understandings.' This narrative, (adds Dr. Combe,) affords a striking confirmation of the truth, that unless the brain be adequately nourished and stimulated by the blood, the mental faculties cannot display that energy which characterises them in opposite conditions."*

In cases of extreme abstinence, the body, not being supported by nourishment from without, preys as it were upon itself; the alimentary canal becomes contracted; the muscular walls close on the empty abdomen; the secretions become depraved, the gastric and pancreatic juices, and even serum of the peritoneum, partially absorbed, and the blood becomes diminished in volume and deteriorated. The first effect of starvation is the disappearance of the fat, the elements of which (carbon and hydrogen) are given off through the skin and lungs, having

* The Physiology of Digestion. By Dr. Andrew Combe, M.D. Edited by James Coxe, M.D. Edinburgh, 1849. p. 108.

served to support respiration. The cellular tissue embedded between the muscles becomes absorbed, the whole body emaciated; "it is not, however, the fat only," says Liebig, "which disappears, but also, by degrees, all such of the solids as are capable of being dissolved. In the wasted bodies of those who have suffered from starvation, the muscles are shrunk and unnaturally soft, and have lost their contractility; all those parts of the body which were capable of entering into the state of motion have served to protect the remainder of the frame from the destructive influence of the atmosphere. Towards the end, the particles of the brain begin to undergo the process of oxidation; and delirium, mania, and death close the scene; that is to say, all resistance to the oxidizing power of the atmospheric oxygen ceases, and the chemical process of eremacausis or decay commences, in which every part of the body, the bones excepted, enters into combination with oxygen. The time which is required to cause death by starvation, depends on the amount of fat in the body, on the degree of exercise, as in labour or exertion of any kind; on the temperature of the air, and, finally, on the presence or absence of water. Through the skin and lungs there escapes a certain quantity of water, and as the presence of water is essential to the continuation of the vital motions, its dissipation hastens death. Cases have occurred in which a full supply of water being accessible to the sufferer, death has not occurred till after the lapse of twenty days; and in one case, life was sustained in this way for the period of sixty days."*

In the midst of all this disorganization and dissolution, the mind frequently retains its self-possession with marvellous perspicuity, and is enabled to recount with painful accuracy the physical sufferings to which it is subjected. Some years ago a German merchant, aged 32, who was depressed by severe reverses of fortune, formed the resolution of destroying himself by abstinence, and with that view repaired, on the 15th September, 1818, to an unfrequented wood, where he constructed a hut of boughs, and remained without food until the 3rd of October following, when he was found by the landlord of a neighbouring public house, still alive, but very feeble, speechless, and insensible. Broth, with the yolk of an egg, was given him, which he swallowed with difficulty and died immediately. In the pocket of the unfortunate man was found a journal written in pencil, singular in its kind, giving a narrative of his feelings, and which may be regarded as a curious psychological fragment. It begins thus: "The generous philanthropist, who shall one day find me here after my death, is requested to inter me; and in consideration of this service, to keep my clothes, purse, knife, and letter-case. I moreover observe, that I am no suicide,

* Animal Chemistry, or Chemistry in its relations to Physiology and Pathology. By Justin Liebig, M.D. Edited by William Gregory, M.D. London, 1843. p. 27.

but have died of hunger, because, through wicked men, I have lost the whole of my very considerable property, and am unwilling to become a burthen to my friends." The next remark is dated September 17, the second day of abstinence : "I yet live; but how have I been soaked during the night, and how cold has it been ! O God ! when will my sufferings terminate? No human being has been seen here for three days—only some birds." The next extract continues: "And again three days, and I have been so soaked during the night that my clothes to-day are not yet dry. How hard is this, no one knows; and my last hour must soon arrive. Doubtless during the heavy rain a little water has got into my throat, but the thirst is not to be slaked with water; moreover, I have had none even of this for six days, since I am no longer able to move from the place. Yesterday, for the first time, during the eternity which, alas ! I have already passed here, a man approached me within the distance of eight or ten paces. He was certainly a shepherd. I saluted him in silence; and he returned it in the same manner. Probably he will find me after my death. Finally, I here protest before the all-wise God, that notwithstanding all the misfortunes which I have suffered from my youth, I yet die very unwillingly; although necessity has imperiously driven me to it. Nevertheless, I pray for it. Father, forgive him, for he knows not what he does. More can I not write, for faintness and spasms, and this will be the last. Dated near Forest by the side of the Goat public house, September 29, 1818. J. F. N."*

In this case, amidst all his physical distress, the unhappy individual was enabled to note down his sufferings until the fourteenth day of abstinence; the sensation of cold and faintness arose obviously from diminished nervous energy, and the thirst, from reduction in the quantity and quality of the blood. "Drink, drink,"—"Water, water," is the last agonizing cry of the poor victim, dying, while he writhes tortured upon the rack.

Having thus far explained the physiological principles which govern the assimilation of the elements of nutrition derived from solid aliments, we are prepared to consider the physiological and psychological effects of those stimulants which, principally in a fluid form, are commonly used as articles of diet.

The universality of the appetite for intoxicating drinks is a curious fact in the history of our species. "It seems," says Dr. Robertson, "to have been one of the first exertions of human ingenuity to discover some composition of an intoxicating quality; and there is hardly any nation so rude, or so destitute of invention, as not to have succeeded in this fatal research."† The juice of the grape was

* Hufeland's Journal. Apud Thackrah. *Op. cit.*, p. 42.

† History of America, vol. i., p. 396. 4to. Edinburgh. Apud Dugald Stewart, *Op. cit.;* Vol. i., p. 18.

undoubtedly the first intoxicating beverage known to mankind; hence Bacchus, after his education by the Nysæan nymphs, is said to have traversed nearly the whole globe, introducing the culture of the vine, and diffusing refinement wherever he went. The Greeks and Romans were certainly ignorant of ardent spirits; but the use of the still was known to Geber in the seventh century, who, in a curious book entitled "Liber Investigationis Magisterii," describes the process of distillation by alembics, "per descensorium et per filtrum." The art of distillation was long preserved in profound secrecy by the alchemists, and it is affirmed by the erudite in these matters, that Raymond Lully, who flourished in the thirteenth century, was the first who gave the name of Alcohol to the spirit of wine so obtained. For two or three centuries after its discovery, alcohol was used only as a medicine; and the physicians of those days, attributing to it the extraordinary property of prolonging life, designated it the *elixir vitæ,* and *aqua vitæ.* Hence, the French still designate their Cognac "*eau de vie.*" Without entering into minute chemical details, the process of fermentation, under its simplest conditions, consists in adding a certain quantity of yeast or other ferment to an aqueous solution of sugar, which, subjected to a certain temperature—say between 70 and 80 Fahr.—resolves itself into carbonic acid, sugar, and alcohol. The carbonic acid is evolved, the sugar, provided a due temperature be maintained, soon disappears, and alcohol is gradually formed. *

Accordingly brandy, rum, gin, whiskey, are the spirituous products obtained by distilling fermented liquors, and most commonly used as articles of exhilarating luxury,† and although they differ very materially

* Brande, *Op. cit.*, p. 1647.

† Brandy is the result of the distillation of wine, and its qualities vary with the kind of wine from which it is obtained, and the precaution with which it is distilled. Its flavour and fragrancy are derived from a portion of essential oil, and of an etherial product, which pass over along with the alcohol and water in the process of distillation. Its average sp. grav. is 0·825 at 60°. It contains about 93 per cent. of alcohol, or 42 per cent. of absolute alcohol. (Sp. grav. 0·791.)

Rum is distilled in the West and East Indies from a fermented mixture of molasses and water, with the skimmings from the sugar boilers, and the lees or spirit-wash of former distillations. Its peculiar flavour is ascribed to an oily product, formed probably in the process of fermentation, to which its sudorific property may probably be in part referred. Its average strength is 53 to 54 per cent. of alcohol (sp. grav. 0·825 at 60°), or about 42 per cent. of absolute alcohol.

Gin, or Geneva—from genievre, juniper—is prepared from different kinds of corn spirit; it was originally largely imported from Holland, and hence known as Hollands, or Holland gin. Its flavour is derived from juniper berries, or the essential oil of juniper, which contributes to its diuretic qualities.

Whiskey, a term said to be derived from the Irish usquebaugh, is also a corn spirit, and when genuine its characteristic flavour is derived from the malt used in its manufacture having been dried over peat or turf fires; but this odour and flavour of burned turf or peat-reek is frequently given to raw corn spirit by impregnating it with peat smoke. The average strength of genuine Scotch and Irish whiskey amounts to about 54 per cent. of alcohol (sp. grav. 0·825 at 60°). Brande, *Op. cit.*, vol. ii., p. 1650.

from each other, and possess very different qualities—inasmuch as brandy is simply cordial and stomachic; rum, heating and sudorific; whiskey and gin, diuretic; yet the stimulating quality of each depends entirely on the quantity of alcohol which enters into its composition. Wines, too, although containing the same absolute amount of spirit, vary materially in their qualities and stimulating effects. To what is this to be attributed? Dr. Paris accounts for it by supposing that, in some wines, the alcohol is not only more intimately mixed with water, but its combination with the extractive matter is more perfect than in other wines; consequently the spirit is incapable of exercising its deleterious effects before it becomes altered in its properties and partially digested. It follows, therefore, that from the peculiar state of the digestive organs, the intoxicating effect of the same wine will vary at different times in the same individual. Hence, also, imperfectly-fermented spirits, apart from any noxious ingredients with which they may be adulterated, will have a very deleterious effect upon the animal economy. Independent, too, of the alcohol itself, their influence will be very much modified by the various acid, saccharine, mucilaginous, and other adventitious matters, which enter into their composition. Hence the practice, so generally adopted in Scotland and Ireland, of dissolving sugar in boiling water before adding the whiskey, is founded upon correct chemical principles, for the condensation between the fluids is hereby greater, and the combination of the spirit with the saccharine matter held in solution more complete. It is also a matter of common observation, that the excitement produced by one kind of wine or spirit differs very materially from another. The wines of Oporto give rise, when drank to excess, to an excitement of a sluggish nature, very different from that derived from sparkling champagne, which intoxicates very speedily, because the small quantity of alcohol which this wine contains rises up along with the bubbles of the carbonic acid gas in a volatile state, producing an excitement of a more lively and agreeable character, and of shorter duration, than that which is caused by any other species of wine.* The different effect which different alcoholic liquors have upon the mind is a matter of common observation; but independent of their direct action on the nervous system, which may be accounted for upon chemical and physiological principles, the peculiar idiosyncracy of each individual mind must be taken largely into account. "It is well known," says Coleridge, "how nearly allied to frenzy are the effects of spirituous liquors on men who have strong feelings and few ideas. The quantity of stimulus which, taken by a man of education, surely as it

* History of Wines, Ancient and Modern. By A. Henderson, M.D. London, 1824. p. 353.

will hasten the decay of all his powers, would yet for the time only call them into full energy,

> "And only till unmechanized by death,

> Make the pipe vocal to the player's breath;"

the same quantity renders an uneducated man a frantic wild beast. Nor do these effects cease with temporary intoxication; but engender habits of restlessness, a proneness to turbulent feelings even when the man is sober; in short, a general inflammability of temper; nor can it be denied, whatever may be its causes, that there exists a certain nationality of constitution, which occasions the poison of spirituous drinks to act with greater malignity in some countries than in others."* The propensities of individuals, and their peculiar habits of thought and feeling, give a corresponding tone to the character of their inebriation—the lover of poetry spouts verses—the orator declaims—the disappointed politician pours forth invectives against his party, whilst he whose mind is demoralized gives way to obscene jesting, profane language, and closes his night's debauch in some such stew of iniquity as Hogarth has depicted in his "Harlot's Progress."

Among the various expedients that have been resorted to for the purpose of abolishing this vice, the establishment of temperance societies was some years ago adopted; and, especially in America and Ireland, have been of essential service; but the prohibition-principle we are afraid scarcely strikes at the root of the evil. Animated by the zealous desire of assisting the "Temperance movement," as it has been called, a worthy individual, Mr. Eaton, early in the year 1848, advertised a prize of one hundred guineas for the best essay on the "Use and Abuse of Alcoholic Liquors in Health and in Disease;" and although we agree with our able contemporary the Athenæum, that the competition for prizes adjudicated upon the shrine of Mammon does not suggest the highest motives for making literary or scientific researches, yet we have been much gratified by the perusal of the two very able essays which have on this occasion been called forth. The successful candidate to whom, we are informed, the prize was unanimously awarded, was Dr. Carpenter; and the next best essay, in the opinion of the umpires, was by Dr. Spencer Thomson; both are published, and the titles of the works are annexed to the present article. Dr. Carpenter had already signalized himself as one of the champions of total abstinence, and, as might be anticipated, he has brought all his scientific knowledge and practical experience to bear, with lawyer-like sagacity, upon his brief, wherefore his evidence and argumentation appear to be impressed with an *ex parte* colouring, which is, in a scientific point of

* Essays on his own Times, forming a second Series of the Friend. By S. T. Coleridge. Edited by his Daughter. 3 vols. London, 1850. Vol. iii. p. 795.

view, to be regretted. The interests of science ought not to be enlisted, in a special-pleading spirit, to uphold in a one-sided manner any popular cause, particularly one that is not founded on the basis of true philososophy; for the abuse of alcoholic liquors no human being would undertake to defend, and to found a prohibition of any article of consumption upon its abuse is very illogical. Dr. Carpenter, therefore, has been arraigned before the bar of the profession for having promulgated views respecting the exhibition of alcoholic stimuli, which are not in accordance with sound principles of physiology and pathology. The essay of Dr. Thomson is certainly written in a more impartial tone; but both contain much valuable information, and they may be considered valuable contributions to medical literature. The prohibition-principle Dr. Carpenter himself is, under certain contingencies, unable to maintain; he is forced, with a reluctant grace, to admit that "alcoholic liquors may be occasionally had recourse to with advantage:" therefore there is here a flaw in his indictment—a broken link in his chain of argument. Far more philosophical would it be to deal with the question in a more comprehensive spirit, and deter people from committing excess, by pointing out the moral and physical evils which every form of intemperance infallibly produces. The deleterious chemico-physiological effects of alcohol on the blood and nervous system, and the lamentable disintegration and ruin which it entails on the intellectual faculties, suggest a more awful warning to the mind of a reflecting man, than the skeleton itself which was introduced as a *memento mori* at the feasts of the Egyptians.

The physiological effects of alcohol on the system have been very carefully investigated, and it is now clearly ascertained that the spirit is absorbed by the blood-vessels of the stomach, and enters into the current of the circulation; "owing to its volatility," says Liebig, "and the ease with which its vapour permeates animal membranes and tissues, alcohol can spread throughout the body in all directions."* We are informed by Dr. Ogsten of Aberdeen, that he examined the body of a woman, aged forty, who had drowned herself when intoxicated; and in addition to the usual appearances found in drowned persons, he and the other medical gentlemen present discovered in the ventricles of the brain four ounces of a fluid, having all the physical qualities of alcohol.† In the Illinois Medical and Surgical Journal, a case is recorded of the examination of a man who was found drowned, after having been drinking many days, and a fluid giving out a strong alcoholic odour was discovered in the ventricles of the brain.‡

* Liebig, *Op. cit.*, p. 239.
† Edinburgh Medical and Surgical Journal, 1833. vol. xl. p. 293.
‡ Medical Times. Vol. xii., p. 320.

Dr. Percy injected a quantity of strong alcohol into the stomach of a spaniel, which immediately died; and afterwards succeeded in obtaining, by distillation, a portion of the spirit from the brain. In order to discover whether drinks are absorbed with the chyle, Magendie made a dog swallow a quantity of diluted spirit with his food; in half an hour afterwards the chyle was examined, which exhibited no trace of alcohol; but the blood exhaled a strong odour of it, and by distillation yielded a considerable quantity.* There can be no doubt, therefore, that when alcohol, whether in the form of wine or spirituous liquid, is introduced into the stomach, it is immediately and rapidly absorbed; but this is not all—it is also clearly proved that, without entering into the circulation, alcohol will, through the medium of the nerves, act directly on the brain, and produce instant death. Orfila relates the case of a soldier who drank eight pints of brandy for a wager, and died instantly.† Only a few years ago a waterman, in attendance upon a cab-stand in the Haymarket, drank, for a bribe of five shillings, a whole bottle of gin at a single draught, and immediately dropped down insensible and died. In some cases death takes place, as Dr. Ogsten well describes, from asphyxia; the increased quantity of blood in the brain produces pressure at its base; the functions of the respiratory nerves thereby become suspended—the motions of the chest are impeded—the lungs congested, and the blood, no longer receiving the chemical changes necessary for circulation, the heart, from the want of its ordinary stimulus, contracts more and more feebly, until its irritability becomes exhausted, and life extinguished.‡ Bearing in mind these very important physiological facts, we must be prepared to expect that the alcoholic elements introduced into the blood, and brought into immediate contact with the tissues of different organs, will derange the functions which they are severally destined to perform, and the amount and character of the mischief so produced will correspond with, and be modified by, the peculiarities of their individual organic structure. With these facts before us, when we consider the delicate structure of the brain, as revealed to us by the progress of microscopic anatomy, we must be prepared for the physical and mental derangement which must arise, either from the alcohol itself, or its elements, being brought into direct contact with the vesicular neurine or granular matter entering into the composition of its white and grey substance. According to our most recent physiological views, the vesicular matter is the source of nervous power, and associated, as the material instrument of the mind, with all its manifestations, whether in the simple exercise of perception, or the more complicated operations of the thinking principle. We are then to conceive the

* Thomson, *Op. cit.*, 43.

† Toxicologie Générale. T. ii. p. 454.

‡ Dr. Ogsten, *loc. cit.*, p. 293.

simple or organic structure dedicated to this high function brought into contact with irritating and noxious elements. The result must obviously be a disturbance in the manifestations of the mind proportioned to the organic derangements so produced, and without, therefore, taking a materialistic view of the changes which take place, the obliteration of some, and the derangement of other of the intellectual faculties, are hereby satisfactorily accounted for. It is certain, that when the circulation in the grey matter of the convolutions is retarded by congestion or accelerated by unwonted stimulation, there is a corresponding state of stupor or mental activity, amounting even to delirium, produced; and, indeed, it has been suggested by some of our most eminent physiologists, that "every idea of the mind is associated with a corresponding change in some part or parts of the vesicular surface."* In the first stage of intoxication, there is arterial acceleration through the brain; the countenance becomes animated, the face flushed, the eye sparkles with brilliancy, and there is a general exaltation of nervous energy. It is now, says the late accomplished Dr. Macnish, that "the thoughts which emanate from their prolific tabernacle are more fervid and original than ever, they rush out with augmented copiousness, and sparkle over the understanding like the Aurora Borealis, or the eccentric scintillations of light upon a summer cloud. In a word, the organ is excited to a high but not a diseased action, for this is coupled with pain, and instead of pleasurable produces afflicting ideas. But its energies, like those of any other part are apt to be over excited. When this takes place, the balance is broken; the mind gets tumultuous and disordered, and the ideas inconsistent and absurd."† Then follows the second stage of intoxication, consequent on excessive stimulation; there is now venous retardation characterized by stupor—languor, drowsiness, obscurity of vision and an incapacity of exercising volition. The intellectual faculties are now completely prostrated. Lastly comes the stage of cerebral compression—the head drops on the chest, the eyes lose their expression, the heart labours, the breathing is stentorious, and a state of coma, more or less complete, succeeds, which not unfrequently terminates in death. In this state some prognosis may be gathered from the condition of the iris. Mr. Bedingfield has made this observation, "If the iris retain its contractile power, the patient will generally recover, however overpowered the senses may be—if, on the contrary, it remain in a state of extreme dilation when a strong light is directed upon it, only a feeble hope of recovery can be entertained."‡

* The Physiological Anatomy and Physiology of Man. By Robert Bentley Todd, M.D., and W. Bowman. 2 vols. London, 1847. Vol. i. p. 364.

† The Anatomy of Drunkenness. By Robert Macnish, LL.D. Glasgow, 1838, p. 110.

‡ Thomson, *Op. cit.*

Independent, however, of the symptoms produced by the disturbed circulation within the brain, Dr. Thackrah conceived that the nervous substance itself possessed some peculiar affinity for alcohol; and Dr. Carpenter attributes a "selective power" as he terms it, to the cerebrum, which may account for the intellectual powers being affected before any disorder of sensation or motion manifests itself.* We do not apprehend that this hypothesis is required to explain these phenomena which are consequent upon over-stimulation, for analogous effects are produced on the nervous system by opium, hashish, tobacco, and other neurotic stimulants.

The fact of alcohol being brought into contact, through the circulation, with the molecular structure of the brain, and other nervous centres, is quite sufficient to account for the series of pathological and psychological changes which terminate in permanent organic lesion and insanity. There can be no doubt, that the increased activity of the circulation through the brain, gives rise, in the first instance, to that rapid flow of ideas which seems to overwhelm the reflective faculties. Opportunities have occurred of observing the effect of increased circulation upon mental phenomena. Sir Astley Cooper relates the case of a young gentleman brought to him who had lost a portion of his skull just above the eyebrow. "On examining the head," says Sir Astley Cooper, "I distinctly saw the pulsation of the brain was regular and slow; but when he was agitated by opposition to his wishes, the blood was directly sent with increased force to the brain, the pulsation became frequent and violent.† Dr. Caldwell relates the case of a young woman who had lost a large portion of her scalp, skull-bone, and dura mater, and a corresponding portion of brain was laid bare, and open to inspection. When she was in a dreamless sleep, her brain was motionless and lay within the cranium; but when her sleep was imperfect, and she was agitated by dreams, her brain moved, and protruded without the cranium, forming cerebral hernia. In vivid dreams, reported as such by herself, the protrusion was considerable, but when she was perfectly awake, especially if engaged in active thought or sprightly conversation, the protrusion was still greater."‡ This increase in the activity of the circulation through the brain, accounts for the intellectual excitement observed, not only in the early stages of insanity, but on the accession of inflammation, and to this cause may perhaps be ascribed the clairvoyance of the dying, so admirably described in the *καυσος* of Aretæus.§

* Dr. Carpenter, *Op. cit.* p. 20.

† Sir Astley Cooper's Lectures on Surgery. Edited by Tyrrel. Vol. i. p. 279. London.

‡ The Principles of Physiology applied to the preservation of Health. By Andrew Combe, M.D. p. 37. Edinburgh, 1841.

§ Essays and Orations. By Sir Henry Halford. London, 1833, p. 81.

In this state of mental excitement, a peculiar lucidity or conscious exaltation of many of the intellectual faculties occurs, which is attended with the most agreeable sensations.

Habitual tippling, or a systematic recourse to intoxicating liquids, gives rise to a chronic form of mental disease, which is characterised by a marked perversion of all the moral feelings. Such persons, without betraying any positive symptoms of drunkenness, are, nevertheless, under the influence of an excitement which produces in them an irritability of temper and a waywardness of disposition, which prompts them to commit acts of indiscretion which frequently become matters of judicial investigation. Was he drunk, or was he sober? He was apparently perfectly collected, and rational; but notwithstanding this, he may have been under the influence of alcoholic excitement, albeit the faculties of the understanding may not manifestly have been deranged. This form of insidious excitement—this state, which is an intermediate condition between sobriety and insobriety—should be carefully watched and guarded against in persons holding responsible situations and positions of trust and danger on board steam-vessels, and having the management of railway engines, &c. In the recent report of the Committee of the House of Commons on the supply and use of spirits in the navy, Captain Drew cites the case of a man who, in a state of excitement, without drunkenness, was continually committing acts of insubordination, for which he was so frequently flogged, that despairing of his reformation the captain determined on invaliding him; but being advised by the surgeon that he was a fine healthy young man, and not a fit subject to be invalided, his allowance of grog was altogether stopped, and the result was, that he became completely an altered man, and not a complaint was made against him while he remained in the ship. Upon cross-examination the captain stated, that "he had never had any suspicion of his being drunk; he never showed any indications of being in liquor," nevertheless, he was "in a state of constant excitement from his allowance, which induced him to do nothing and constantly commit acts of disobedience."* Captain Hamilton, in the same report, says, "that drunkenness is the cause of nearly all the punishments on board a ship is so well known that I need not repeat what has so often been said; but I would go further, and add, that where people do not even get drunk, habitual spirit-drinking produces an irritability of temper in the seamen and the officers that is the cause of much evil in the daily routine of duty."† This form of mental disease frequently terminates in homicidal insanity; unless apoplexy, paralysis, or delirium tremens, supervene, and

* Report of the Committee appointed to Inquire into the supply of Spirits in the Navy. 1850, p. 13.

† Ibid. p 20.

lead to a fatal result. The case is still fresh in the recollection of the public of a captain of a merchant-vessel, who, suffering under this form of disease, wounded and cruelly maimed many of his crew, and who is now an inmate of Bethlehem Hospital.

The practice of taking opium to exhilarate the spirits is not, we have reason to believe, so common now in this country as it was some years ago. Fashion controls even vice in polished society. When it became whispered abroad, that some few eminent statesmen and literary men of distinction indulged occasionally in this pernicious habit, even ladies moving in the higher circles of the aristocracy imagined that this "fatal drug" must possess some Circean charm, and had recourse to it for the purpose of enabling them to sustain the excitement necessary for going through the fatigue of midnight routs, and parties succeeding each other the same night with dazzling rapidity. The publication of that charming and very remarkable work, "The Confessions of an English Opium-Eater," contributed rather to extend than suppress the pernicious practice; for, albeit the accomplished author depicted in fearful colours the "pains of opium," yet the pleasures and transcendental happiness which he describes himself to have experienced during his reveries,—the visions which opened before him,—the apocalypse of the spiritual and inner world, are described with such fascinating eloquence, that his voice of warning was in reality that of the tempter, inviting others to participate in his mystic and dread enjoyment. We ourselves knew many young men who injured their health by having, experimentally, recourse to it, forgetting entirely that the character of the excitement so produced, must depend on the mental idiosyncrasy and previous habits of thought of every individual. "If a man," observes Mr. De Quincey, "whose talk is of oxen, should become an opium-eater, the probability is, that (if he is not too dull to dream at all) he will dream about oxen;" so, too, it is not to be anticipated that Oriental imagery,—the pagodas of China,—the temples of Isis and Osiris,—the domes and cupolas of Jerusalem,—and the solemn music of anthems celebrating religious festivals, will crowd upon a mind which is habitually unimaginative. We regard the "Confessions of an English Opium-Eater" as a psychological romance, although it be a true autobiographical account of those mental phenomena which occurred to a mind peculiarly constituted, accustomed in its waking state to habits of profound thought, and commanding a vast store of ancient and modern learning, tinctured with a certain degree of mysticism derived from the school of Kant, Fichte, and other German philosophers. We may truly say, that Mr. De Quincey is one of the most remarkable men we ever had the pleasure of meeting; his conversation is always characterised by the clearest reasoning and the happiest choice of language; he is a profound Greek scholar, and his erudition extends through the history of all countries; few men are better acquainted with Eastern literature, and, although it is some five-and-twenty years since we were in the habit of frequently meeting him, it gives us unfeigned satisfaction to learn that he has entirely given up the use of opium, and is in the enjoyment of excellent health.

The psychological effects of alcoholic or vinous potations differ materially from those arising from the use, or rather, the abuse of opium.

"The pleasure given by wine," we quote the 'Confessions of an Opium-Eater,' "is always mounting and tending to a crisis, after which it declines; that from opium, when once generated, is stationary for eight or ten hours: the first, to borrow a technical distinction from medicine, is a case of acute, the second, a case of chronic pleasure; the one is a flame, the other a steady and equable glare. But the main distinction lies in this, that whereas wine disorders the mental faculties, opium, on the contrary (if taken in a proper manner) introduces amongst them the most exquisite order, legislation, and harmony. Wine robs a man of his self-possession, opium greatly invigorates it; wine unsettles and clouds the judgment, and gives a preternatural brightness and a vivid exaltation to the contempts and admirations,—the loves and the hatreds of the drinkers; opium, on the contrary, communicates serenity and equipoise to all the faculties active or passive; and, with respect to the temper and moral feelings in general, it gives simply that vital warmth which is approved by the judgment, and which would probably always accompany a bodily constitution of primæval or antediluvian health * * * *. Wine constantly leads a man to the brink of absurdity and extravagance, and beyond a certain point it is sure to volatilize and to disperse the intellectual energies; whereas, opium always serves to compose what had been agitated, and concentrate what had been distracted."*

To counterbalance, however, these apparent advantages of the effects of opium over wine, opium-eating may be described as, strictly speaking, "a solitary vice."

"The opium-eater," observes our author, "naturally seeks solitude and silence as indispensable conditions of those trances or profoundest reveries which are the crown and consummation of what opium can do for nature;" but even in this state of sublunary bliss, it is confessed that, "his intellectual apprehension of what is possible infinitely outruns his power, not of execution only, but even of power to attempt: he lies under the weight of incubus, and night-mare; he lies in sight of all that he would fain perform, just as a man forcibly confined to his bed by the mortal languor of a relaxing disease, who is compelled to witness injury or outrage offered to some object of his tenderest love—he curses the spells that chain him down from motion;—he would lay down his life if

* Confessions of an English Opium Eater. London, 1826. p. 95.

he might get up and walk—but he is powerless as an infant, and cannot attempt to rise."*

A state very analogous to this is produced by various preparations of the Indian hemp—the Cannabio Indica, known under the name of Hashish, from which are prepared Sidhee or Bang, Majoon, and several forms of extract.†

In a very interesting work recently published by Mr. Urquhart, the psychological effects of this drug are thus described : "The first time I took it was about seven in the morning, and in an hour and a half after I perceived a heaviness of the head, wandering of the mind, and an apprehension that I was going to faint. I thence passed into a state of half trance, from which I awoke suddenly and was much refreshed. The impression was that of wandering out of myself. I had two beings; and there were two distinct and concurrent trains of ideas. Images came floating before me—not the figures of a dream, but those that seem to play before the eye when it is closed, and with those images were strangely mixed the sounds of a guitar which was being played in an adjoining room; the sound seemed to cluster in, and pass away with the figures on the retina. The music of the wretched performance was heavenly, and seemed to proceed from a full orchestra, and to be reverberated through long halls of mountains. These figures and sounds were again connected with metaphysical reflections, which also like the sounds clustered themselves into trains of thought, which seemed to take form before my eyes, and weave themselves with the colours and sounds. I was following a train of reasoning, new points would occur and concurrently. There was a figure before me throwing out corresponding shoots like a zinc tree; and then, as the moving figures reappeared, or as the sounds caught my ear, the other classes of figures came out distinctly, and danced through each other."‡ The effect of irregular and hurried circulation through the brain, and its immediate connexion with these trains of thought, are here plainly indicated; so much, however, has recently appeared on the effects of Hashish § that we need only point out the fact, that all these neurotic stimulants act upon the same principle; they affect the mind through the medium of the circulation.

Considered under the head of mental dietetics, the question recurs

* Ibid. p. 156.

† Pharmaceutical Journal and Transactions. Edited by Jacob Bell, 1845. Vol. v. p. 83.

‡ The Pillars of Hercules, a Narrative of Travels in Spain and Morocco, in 1845. By David Urquhart, Esq. 2 vols. London, 1850. Vol. ii. p. 129.

§ Du Hashish et de l'Aliénation Mentale, par J. Moreau. Paris, 1845. British and Foreign Medical Review. By John Forbes, M.D. Vol. xxiii. 1847.

under what circumstances they ought to be had recourse to, and whether as articles of diet, all alcoholic and vinous stimulants may not be dispensed with. There can be no doubt that opium and hashish should in this country be had recourse to only as medicinal agents, and even then they should be administered with great care. The concurrent testimony, however, of the most approved authors on diet, we allude to Dr. Wilson Philip, Dr. Paris, Dr. Pereira, Dr. Mayo, Dr. Andrew Combe, is in favour of alcoholic and vinous stimulants being occasionally used with moderation.

---

